# Psychoeducation to facilitate return to work in individuals on sick leave and at risk of having a mental disorder: protocol of a randomised controlled trial

**DOI:** 10.1186/1471-2458-14-1288

**Published:** 2014-12-17

**Authors:** Pernille Pedersen, Hans Jørgen Søgaard, Bjarne Frostholm Yde, Merete Labriola, Ellen A Nohr, Chris Jensen

**Affiliations:** Psychiatric Research Unit West, Regional Psychiatric Services West, Central Denmark Region, Gl. Landevej 49, 7400 Herning, Denmark; Institute of Clinical Medicine, University of Aarhus, Aarhus, Denmark; Public Health and Quality Improvement, Central Denmark Region, Aarhus, Denmark; Regional Psychiatric Services, Central Denmark Region, Silkeborg, Denmark; Section of Clinical Social Medicine and Rehabilitation, School of Public Health, University of Aarhus, Aarhus, Denmark; Institute of Clinical Research, University of Southern Denmark, Odense, Denmark; Department of Public Health and General Practice, Norwegian University of Science and Technology, Trondheim, Norway; National Centre for Occupational Rehabilitation, Rauland, Norway

**Keywords:** Sickness absence, Psychoeducation, Mental health, Return to work, Psychological symptoms, Mental health-related quality of life, Locus of control

## Abstract

**Background:**

Sickness absence due to poor mental health is a common problem in many Western countries. To facilitate return to work, it may be important to identify individuals on sick leave and at risk of having a mental disorder and subsequently to offer appropriate treatment. Psychoeducation alone has not previously been used as a return to work intervention, but may be a promising tool to facilitate return to work. Therefore, the aim of the study is to evaluate the effectiveness of psychoeducation designed specifically to facilitate return to work for individuals on sick leave and at risk of having a mental disorder. The psychoeducation was a supplement to the various standard offers provided by the job centres.

**Methods/Design:**

The study is a randomised controlled trial, in which individuals on sick leave either receive psychoeducation and standard case management or standard case management alone. Participants were individuals with mental health symptoms, who had been on sick leave from part-time or full-time work or unemployment for about 4–8 weeks. The psychoeducational intervention was group-based and the course consisted of 2 hour sessions once a week for 6 weeks. The course was given by psychiatric nurses, a psychologist, a social worker, a physiotherapist and a person who had previously been on sick leave due to mental health problems. The sessions focused on stress and work life, and the purpose was to provide individuals on sick leave the skills to understand and improve their mental functioning.

The primary outcome is the duration of sickness absence measured by register data. Secondary outcomes include psychological symptoms, mental health-related quality of life, and locus of control. These outcomes are measured by questionnaires at the start of the intervention and at 3 and 6 months follow-up.

**Discussion:**

On the basis of this trial, the effect of psychoeducation for individuals on sick leave and at risk of having a mental disorder will be studied. The results will contribute to the continuing research on sickness absence and mental health. It will primarily show whether psychoeducation can lead to faster and sustainable return to work.

**Trial Registration:**

Clinical Trial.gov NCT01637363. Registered 6 July 2012.

## Background

### Sickness absence and mental health problems

In many Western countries, mental health problems are a main cause of sick leave [1–3]. Common mental disorders, such as adjustment disorders, depression, anxiety and somatoform disorders constitute the most prevalent causes of long-term sickness absence [4–6]. Interventions aiming to facilitate return to work (RTW) for this group have received attention in recent years and a review by Soegaard et al. showed that several research papers have been published [7]. Moreover, two Cochrane reviews have described that a broad range of interventions have been tested, such as pharmacotherapy, relaxation therapy, exercise programmes, occupational therapy, enhanced primary care, employee assistance programmes and psychological interventions [2, 8]. Psychological interventions, such as cognitive behavioural therapy and problem-solving therapy, are commonly used [2]. In this study, the effect of a psychological intervention will be tested, i.e. psychoeducation (PE), in individuals on sick leave and at risk of having a mental disorder. PE has been chosen as it is a simple intervention, which can convey knowledge of personal mental health problems to a broad range of individuals on sick leave. These acquired competences will presumably be helpful in the RTW process. To our knowledge, the effect of PE on RTW has not yet been evaluated; however, evaluations have been recommended [9].

### Psychoeducation

PE is education offered to individuals with mental disorders or mental distress and can include their relatives [10, 11]. The purpose of PE sessions is to provide individuals with tools that enable them to be more active in their recovery process and to cope with their situation [12]. Thus, psychotherapeutic techniques, such as behavioural activation, cognitive behavioural therapy and problem-solving therapy, are often included [13]. These techniques aim to promote awareness and proactivity in relation to recognition of episode recurrences, to change the individuals’ behaviours and attitudes towards their disorders as well as to improve psychosocial and occupational functioning plus quality of life [14, 15].

In this trial, PE is used as a group-based intervention; however it can be applied in a variety of formats [11]. The number of sessions varies, but many psychoeducational interventions include 6–12 sessions [13, 16–22].

PE, in combination with standard pharmacotherapy, has proven to have a long-term effect (for up to 5 years) in terms of reducing the number of recurrences and prolonging the time to recurrence in individuals who suffer from depression or bipolar disorder [16, 22, 23]. Additionally, PE can reduce manic and depressive symptoms for up to 1 year after the intervention [13, 16–19, 22, 24] as well as prevent depression in individuals with subclinical depressive symptoms [19]. Participants with a relatively mild initial depressive symptomatology seem to benefit more from the education than participants with higher levels of initial symptoms [13, 19, 25]. PE has also proven effective in terms of non-clinical outcomes. Within 3 months after the intervention, PE has shown to be effective in increasing participation in pleasant activities, social interaction [26], self-esteem [24, 26] and the frequency of seeking social support [26]. These outcomes are presumably all important for RTW.

### Information and education in RTW-interventions

To our knowledge, PE alone has not previously been used as an RTW intervention. However, information and education on mental health problems have been used in combination with other types of interventions. These interventions have mainly included individuals on sick leave or employees suffering from stress or work-related stress [4, 27–32]. De Vente et al. [28] and Willert et al. [31] implemented PE taught by a psychologist as part of their intervention; but, the content was not further elaborated. Information and advice on lifestyle, coping, wellness, health, nutrition, physical exercise and preparation of RTW have been employed in many studies [4, 28–30]. Furthermore, in the study by Stenlund et al. [4] the relatives were invited to participate in part of the intervention.

In general, interventions comprising information and education to stressed individuals have not resulted in better RTW outcomes for the intervention group than for the control group. Nevertheless, the study by van der Klink et al. [32] found a higher RTW rate in the group receiving information. The interventions by Willert et al. [31] and Grossi et al. [29] were able to lower the scores on depression, burnout and perceived stress in the intervention group. Grossi et al. concluded that a course teaching patients to identify, understand and handle stress symptoms may be more effective in reducing stress-related exhaustion than conventional treatment alone [29]. One reason for the overall limited effect could be that many of the studies within the field had low power, as commented by others [4, 33]. As a general rule, the required sample size in studies measuring occupational outcomes should be larger than the sample size in studies measuring clinical outcomes alone [8].

### Sickness absence in a Danish context

In Denmark, social workers in the municipal case management centres, the so called “job centres” administrate sickness benefit cases and are part of the initial RTW process of individuals on sick leave. The first consultation between the social worker and the individuals on sick leave must take place before the end of the first eight weeks of absence [34]. The social workers may require a workability record from the general practitioners, but this may not be obtained until after the first consultation. Thus, the social workers often rely on the information from the beneficiaries, for instance regarding their diagnoses [35]. The social workers do not screen for mental health symptoms. However, it may be advantageous to screen and to identify individuals at risk of having a mental disorder as 24% of individuals on long-term sick leave have been assessed to suffer from an undetected mental disorder [36]. Sogaard & Bech have developed a simple screening instrument, SCL-8 AD, to identify individuals at risk of having a mental disorder in the group of individuals on long-term sickness absence (>8 weeks) [36]. The screening instrument is meant as a useful tool for social workers to better identify mental health problems and to offer a tailored rehabilitation strategy.

In a Danish context, there is a lack of evidence-based RTW interventions [9], and the activities offered by the job centres are not necessarily targeted at individuals at risk of having a mental disorder. As a consequence, we intended to evaluate a pragmatic intervention targeted at this population and based on a model which is simple to implement in the Danish job centres.

The intention was to identify individuals on sick leave and at risk of having a mental disorder (screened by SCL-8 AD) and subsequently to offer PE. The study was a pragmatic randomised controlled trial (RCT) testing the intervention in a heterogeneous group of individuals on SA.

### Study aim and hypothesis

The aim of the study was to evaluate the effect of psychoeducation targeted specifically to facilitate RTW as adjunct to standard case management for individuals on sick leave and at risk of having a mental disorder.

It was hypothesised that individuals who participated in the psychoeducational programme would have shorter sickness absence periods compared to the control group, and furthermore, fewer psychological symptoms, improved mental health-related quality of life and internal locus of control.

## Methods/Design

### Study design

In this RCT the intervention group received PE in addition to usual care whereas the control group only received usual care. In Denmark, compulsory activities are provided by the municipal job centres, the purpose being to promote RTW. These activities were considered as usual care.

### Setting

The study was conducted in four municipalities in the Western part of Denmark (Skive, Struer, Lemvig and Holstebro) with a total of approximately 150,000 citizens. The recruitment of participants started in September 2012 and ended in January 2014.

In the spring of 2012, a pilot study was conducted.

### Recruitment

Individuals on sickness absence benefit for about 4–8 weeks were identified weekly during the recruitment period and mailed information about the study, an invitation, a screening questionnaire with inclusion, and exclusion criteria and a return envelope. A reminder to return the questionnaire was sent after 10–14 days. The screening questionnaire included the questionnaire SCL-8 AD. It consists of 13 questions derived from SCL-92 and has been evaluated to detect mental disorders (especially depression, anxiety and somatoform disorders [37]) in individuals on long-term (>8 weeks) sickness absence. A cut-point of ≥5 was chosen for inclusion, with a sensitivity of 75%, a specificity of 68% and a positive predictive value of 51% [37]. The instrument has previously been used in a larger Danish national RTW project [38].

Eligible individuals were contacted by phone by a research assistant who gave information about the study. If they agreed to participate in the study, they were randomised. Subsequently, they were mailed information about their allocation and a consent form to fill out and return.

Individuals could only be invited to participate in the study once during the study period.

### Participants

The target population were individuals on sick leave from part-time or full-time work or unemployment.

Participants were eligible for the study if they were between 18 and 64 years old and had a SCL-8 AD score ≥5.

Participants were ineligible when they met one or more of the following exclusion criteria: 1) did not communicate in Danish; 2) had been on sick leave due to mental health problems for more than 3 consecutive months during the preceding year; 3) were pregnant; 4) had a supported job/were in job training/in rehabilitation/had retired.

**Figure 1:**
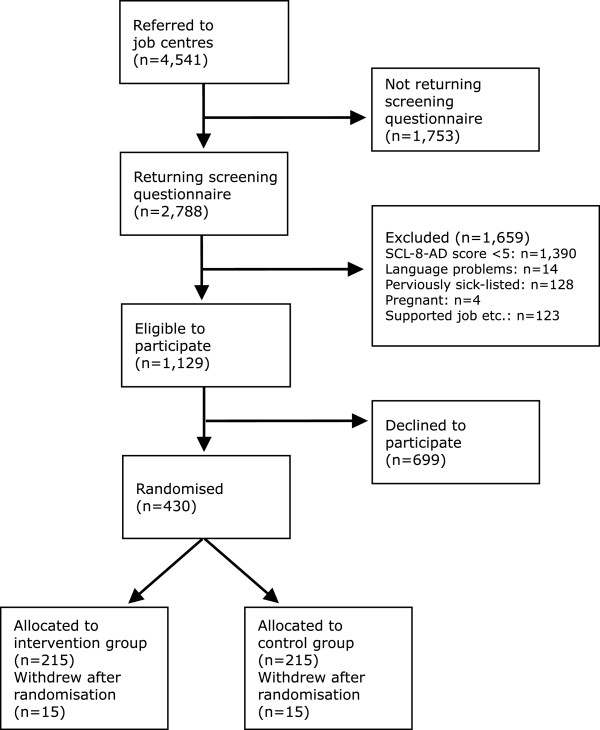
**Flowchart of the study.**

A total of 4,541 individuals were on sick leave and referred to the job centres in the study period. Of the 1,129 eligible individuals, 430 accepted to participate (Figure 1). After randomisation, 30 participants withdrew from the study. RTW data were registered for all participants.

### Randomisation

The participants were equally randomised (1:1) to one of two parallel groups; the intervention group or the control group. The study was designed as a superiority trial. A computerised random number generator with a block size 4 was used to allocate participants. The programme was prepared by a data manager with no further involvement in the study. The randomisation was carried out by a research assistant who also informed the participants by mail of their allocation.

### Blinding

The social workers at the job centres were in contact with all study participants to provide the usual social services at the job centre, but they were not informed about their allocation in the study. Due to the nature of the intervention, neither participants nor staff could be blinded to the allocation.

### Baseline and outcome measures

From the screening questionnaire, information on gender, age, education, employment, reason for sickness absence and self-reported recovery expectations (estimation in percentage regarding the probability of being back to work in 6 months) was received.

The records from the job centres were used to retrieve information on whether the participants were fully or partially on sick leave and whether their job situation before sickness absence was full-time or part-time work or unemployment.

At the start of the intervention and at follow-up after three and six months, the participants received a questionnaire by either e-mail or mail. This questionnaire consisted of psychological symptoms (six scales from the Symptoms Checklist 90-R (SCL90-R)) [39], mental health related quality of life (four scales from The 36-item Short Form Health Survey (SF-36)) [40] and Multidimensional Health Locus of Control (MHLC) [41]. A reminder to return the questionnaire was sent after 10–14 days. The participants received a gift certificate of 13 euros for completing each questionnaire.

### Monitoring for participants’ compliance

Questions about attendance in other RTW activities offered by the job centres or co-interventions, such as treatment by the general practitioner, a psychologist or a psychiatrist, were included in the questionnaire three months after randomisation.

The attendance in the PE sessions was registered to monitor compliance.

### Primary outcome

#### Sickness absence duration

Time to full RTW was the primary outcome of the study and was measured by register data from the job centres. It was defined as the period (in days) between randomisation and to *full-time* RTW for at least 4 weeks without (partial or full sickness absence) recurrence. Full return to work was operationalised as not receiving sickness benefits.

### Secondary outcomes

#### Sickness absence duration and recurrence

Time to first RTW was defined as the period (in days) between randomisation and to *first* (partial or full-time) RTW or being fit-for-duty if unemployed for at least 4 weeks without (partial or full sickness absence) recurrence. Thus, the participants could still receive partial sickness benefits.

Furthermore, recurrence of sick-leave was measured, regardless of reason. Time to recurrence was defined as the period between the date of full RTW and the date of recurrence.

The observations were based on register data from the job centres.

#### Psychological symptoms

Six scales from the Danish version of the Symptom Checklist-90-Revised (SCL-90-R) were used to assess psychological symptoms of psychopathologic status [42]. The scales were somatisation, obsessive-compulsive, interpersonal sensitivity, depression, anxiety and phobic anxiety. It is a self-report instrument, and the participants are asked to state how much discomfort, as described in each item, they had experienced during the past seven days. The discomfort is measured on a five-point rating scale ranging from “not at all” (0) to “extremely” (4).

#### Mental health-related quality of life

The four psychologically based scales from the Danish version of The 36-item Short Form Health Survey (SF-36) were used to measure mental health-related quality of life [43]. These domains were vitality, social functioning, role limitation due to emotional problems and mental health. The score of each domain ranges from 0 to 100; the higher the scores, the higher the levels of functioning. Furthermore, the question “In general, would you say your health is” with the options; excellent, very good, good, fair and poor, was included.

#### Locus of control

The Multidimensional Health Locus of Control (MHLC) scale Form C was used to measure health locus of control and can be defined as the degree to which individuals believe that their health is controlled by internal or external factors. The Form C is condition-specific and can be used when studying individuals with an existing health/medical condition. It consists of four subscales: “doctors” and “other people” with each three items, and “chance” and “internal” with each six items. For each item, a Likert-type scale ranging from 1 to 6 was applied (1 representing “strongly disagree” and 6 representing “strongly agree”).

This study applied a Danish version of the questionnaire. It has been translated and back-translated by a person with experience within the field. The translation was made especially for this study. It was tested among participants in the pilot study.

### Treatments

#### Usual care

All the participants received usual care which entailed RTW activities arranged by the job centres. RTW activities typically comprise fitness workout, stress- and pain- management and gradual RTW. The Danish sickness benefit law does not specify which kind of activities should be available. Consequently, a large variation across municipalities is seen in what is being done, when and for whom [44]. Because of the naturalistic study setting, all participants were free to engage in any other treatment as well.

#### Psychoeducation intervention

The PE used in this study was group-based, and the course consisted of 2 hour sessions once a week for 6 weeks. The course was in line with a slow-open group, meaning that new participants could be included shortly after they had accepted to participate. Receiving the intervention as fast as possible had a high priority. All courses were held at two different job centers; two locations were chosen to reduce transportation. Mileage allowance (0.27 euro/km) was offered to the participants.

The courses were conducted and taught by four psychiatric nurses, a psychologist, a social worker, a physiotherapist and a person who had previously been on sick leave due to mental health problems. Two meetings were held to discuss the content of the sessions, and subsequently the teachers prepared the materials. The psychiatric nurses were experienced in PE, and one of them was present at each session. The sessions focused on stress and work life and consisted of a mixture of didactic lectures and group discussions. The purpose was to provide the individuals on sick leave with qualifications to understand and improve their own situation through knowledge, dialogue and personal experiences. The Stress-Vulnerability Model [45] was used to help the individuals recognise sources of stress in their lives and how to eliminate some of them. Moreover, problem-solving techniques and coping strategies were incorporated. The focus was, to a high extent, on the general discomfort which the symptoms caused in everyday life and in particular on handling a job. To a low extent, focus was on diagnosis. The intervention was standardised, and each session followed structured slides to uniform the intervention. Hand-outs were given to the participants. The content of each session is described in Table 1. A session for relatives was included with the purpose of providing them with tools to support the individuals on sick leave. Research has established that when family members benefit from PE, patients experience lower rates of relapse, longer time intervals between episodes, a better treatment adherence and a reduction in symptoms [25]. One session was devoted to a person with a previous sickness absence. People with personal experience may be in a better position than clinicians to give advice and to identify and address psychosocial issues as it is grounded in experimental knowledge and actual feelings [46]. One session on physical exercise was included since studies show that engaging in regular physical activities can improve recovery from mental illness [47, 48].

**Table 1:** **Session-by-session outline for the psychoeducation intervention**

Session	Teachers/faciliteters	Content
1	Psychiatric nurse	• Information on symptoms of stress, depression, anxiety and functional disorders related to the cause of the disorders and the consequences for the ability to work. The teaching focused on diagnoses to a lesser extent than traditional PE. Instead, emphasis was on the general discomfort and functioning in everyday life caused by the symptoms and in particular on handling a job. The session was based on the Stress-Vulnerability Model
2	Psychiatric nurse	• Information on options and appropriate coping strategies related to the mental symptoms and the sick-listing of the participants. The teaching focused on self-awareness, warning signs and lifestyle. The participants were introduced to different cognitive tools, which they could use in their everyday life. The session was based on the Stress-Vulnerability Model
3	Social worker/Psychiatric nurse	• On the basis of the sick-leave legislation, the participants received counselling related to their sick-listing. The teaching provided the participants with tools to facilitate labour market participation and RTW.
4	Psychologist/Psychiatric nurse	• Information on mental reactions and symptoms related to being on sick leave. The teaching provided the participants with tools to achieve a higher level of mental well-being and to facilitate RTW. The participants were informed about where to turn for support and, additionally, psychological challenges and barriers related to RTW were discussed.
5	Physiotherapist/Psychiatric nurse	• The participants were informed about the importance of exercise for health in general and about the influence of exercise on mental well-being in particular. Additionally, training advice and counselling to ensure a continued motivation were given.
6	A person previously on sick leave/Psychiatric nurse	• Both participants and relatives attended the first part of the session which consisted of a presentation by a person who previously had been on sick leave due to mental health problems. The speaker described the course of illness, the process of dealing with personal issues, and the course towards RTW. Subsequently, the participants shared mutual experiences as well as experiences with the speaker. Concurrently, the relatives participated in a session held by a psychiatric nurse. The purpose was to strengthen the abilities of the relatives; in part to support the individuals on sick leave towards RTW, and in part to take their own lives in their hands. The relatives were informed about the symptoms of stress, depression, anxiety and functional disorders.

#### Sample size calculation

Duration of sickness absence until full RTW was chosen as the primary outcome measure and used for sample size calculation. Based on data from a Danish sickness absence study [36], we assumed that 70% would return to work within 6 months (“fail probability” of 0.70). We expected a 40% higher rate of RTW in the intervention group than in the control group, corresponding to a hazard ratio of 1.4. Sample size calculation, using a two-sided significance level of 5% and a power of 80%, indicated a minimum of 397 participants divided equally into the two groups. We decided to include an additional 10% to compensate for drop outs.

### Statistical analysis

It will be studied whether the participants differ from the eligible individuals who declined participation, and if the participants at follow-ups are different from the baseline population in relation to socio demographic and health characteristics. Adherence to the intervention will be described.

The rates of sustainable RTW will be compared between the intervention group and the control group during the first 3 and 6 months after randomisation by means of the pseudo value method [49, 50]. Any effects on psychological symptoms, mental health-related quality of life, and locus of control will be measured in secondary analyses. In those analyses symptoms of depression and anxiety will be the main outcome.

The analyses will be performed using STATA 11 IC (Stata Corp, College Station, TX).

All analyses will primarily be performed on an intention-to-treat basis; however, per-protocol analyses will also be performed [51].

### Ethical considerations

All participants were offered treatment as usual according to their individual needs, i.e. RTW activities offered by the job centres and treatment from health professionals. Participation was voluntary, and project information was given both verbally and in writing. The participants were informed about their rights to decline participation and to withdraw with no consequences in terms of their sickness absence benefits.

Previous research has not indicated that PE induces risk to the participants. However, it has been discussed whether information about possible mental symptoms can implant expectations of pathology and dysfunction [10]. Compared to traditional PE, the intervention in this study focused on diagnosis to a less extent. Therefore, we expect negative expectations of pathology and dysfunction to be rare.

During the sessions, the psychiatric nurses were aware of the participants’ reactions, and, if needed, they talked to them. If the psychiatric nurses observed a need for additional treatment, they could encourage the participants to see their general practitioner or refer them to a psychiatrist (HJS).

All participants were assigned an identification number and were treated anonymously in all analyses. Papers and electronic documentation with names and personal identification numbers were stored securely in locked cabinets or on a password-protected computer.

The study has been notified to and approved by the Danish Data Protection Agency (http://www.datatilsynet.dk). According to the Danish National Committee on Biomedical Research Ethics (written communication), the intervention did not need ethic approval as it did not include biomedical research. The study is registered at Clinical Trials.gov (NCT01637363).

## Discussion

This trial will evaluate the effect of PE on RTW among individuals on sick leave and at risk of having a mental disorder. We will assess the impact of the intervention on sickness absence duration, psychological symptoms, mental health-related quality of life and locus of control.

The study will assess the effectiveness rather than the efficacy of the RTW intervention. Thus, it will evaluate what is possible in practice. As a consequence, participants were included based on a simple screening instrument which is easily applicable for the social workers. Not all the participants may have a mental disorder, meaning that the included individuals can be very different with some suffering from a major depression and some having distress. On the other hand, if individuals on sick leave with a specific diagnosis had been included, then the participants had to be screened by their general practitioner, which deviates from usual practice in the job centres.

Individuals who had been on sick leave due to mental health problems for more than three consecutive months during the preceding year and those without a paid job were excluded. This was done based on the assumption that the intervention would probably not fully accommodate the needs of these individuals. Furthermore, a previous Danish study conducting a psychiatric examination found that the feedback and information based on the examination was most effective for individuals on sick leave from full-time work and without a psychiatric sick leave diagnosis [52].

### Psychoeducation

The topics in the PE course should be versatile to address all the different needs of this heterogeneous group. Consequently, different health professionals were used to provide the individuals with broad information. PE has been documented to be meaningful in settings where a multidisciplinary team effort is available [12]. The psychiatric nurses were highly experienced in PE while the social worker, the psychologist and the physiotherapist had experience in working with individuals on sick-leave. PE can be administered by therapists from various disciplines without extensive training [12]. The use of different health professionals may also be important to avoid that the effect may be ascribed to the influence of a personality of a single professional, which cannot be replicated in other settings.

We decided to provide the courses as a slow-open group and not as a closed group. To our knowledge, PE has not previously been carried out in this way. We chose this setting to be able to offer the intervention as fast as possible since it has been documented to be important [9]. If closed groups had been used, participants could have waited up to 6 weeks to start the intervention. It is plausible that the waiting time could worsen their symptoms. To compensate for the weekly inclusion of new participants, the same psychiatric nurse was present for six successive sessions. She welcomed new participants and was familiar with the group.

### Strengths and limitations

The main strengths of this study are the randomised controlled design and the large sample size. The study includes a sample from a large heterogeneous population which should further a generalisation of our results to individuals on sickness absence in Denmark. Based on registers on sickness benefits, information on all individuals on sickness absence benefits in the source population were retrieved and thus, the study is not affected by incomplete coverage. The risk of bias related to group allocation is low since randomisation was performed by a computerised random number generator. To measure RTW, register data will be used, which is preferable compared to self-report in regard to receive more accurate information on the sick leave period [53]. To our knowledge, this is the first study to include measures of locus of control in this population. First of all, this assessment enables us to describe the external and internal locus of control of the individuals and then to assess whether it changes after PE. This particular questionnaire has not been validated in a Danish context; however, it has been translated, back-translated and pilot-tested in a group of individuals on sickness absence benefits.

The main weakness of this study is that the social workers were not effectively blinded. In collaboration with the individuals on sickness absence benefit, they assess whether the individuals are ready to RTW. About three months after the randomisation, we asked the social workers to guess what group they think the participant belongs to. Their guesses will show whether they have been aware of the group allocation. When examining the effect of an RTW intervention, such as PE, it is not possible to blind the participants or the staff, which may induce bias.

The results will contribute to the continuing research on sickness absence and mental health problems. It will primarily show whether PE can lead to faster and sustainable RTW and enable politicians and leaders of the job centres to decide whether the intervention should be implemented. Results will be available at the end of 2015.
